# Effort-Reward Imbalance, related risk factors and the association with depression and suicidal ideation in German dentists- BMC Psychology

**DOI:** 10.1186/s40359-026-05490-6

**Published:** 2026-08-31

**Authors:** Antonia Geupel, Patricia Gaillard, Rainer Haak, Heide Glaesmer

**Affiliations:** 1 Department of Medical Psychology and Medical Sociology, University of Leipzig, Philipp-Rosenthal-Str. 55, 04103 Leipzig, Germany; 2 Dental Health Center Amberg, Amberg, Germany; 3 Department of Cariology, Endodontology and Periodontology, University of Leipzig, Leipzig, Germany

**Keywords:** Depressive symptoms, Suicidal ideation, Effort-reward imbalance, Risk factors, German dentists

## Abstract

**Background:**

Dentistry involves substantial physical, psychological, and organizational demands. Increasing administrative tasks and structural changes in dental health care may exacerbate imbalances between professional effort and rewards. According to the Effort-Reward Imbalance (ERI) model, such imbalances are linked to adverse mental health outcomes. This study investigates determinants of ERI among German dentists and its association with current depressive symptoms and suicidal ideation.

**Methods:**

A nationwide cross-sectional online survey was conducted in Germany from July 2021 to February 2022, and 589 practising dentists participated in the survey. ERI was assessed using a validated self-report questionnaire. Depressive symptoms were measured with the Patient Health Questionnaire-2, and suicidal ideation with item 9 from the Patient Health Questionnaire-9. Sociodemographic, family-related factors and occupational variables were collected via self-report. Differences between male and female participants were analyzed using chi-square tests and t-tests. A hierarchical linear regression analysis was used to identify predictors of ERI, and Spearman correlations examined associations with current suicidal ideation and depressive symptoms.

**Results:**

The mean ERI ratio was 1.1, with 53.1% of participants showing an unfavorable imbalance. Depressive symptoms were identified in 21.1% of respondents, and 11.7% reported suicidal ideation in the last two weeks. Higher imbalance was significantly associated with increased depressive symptoms and suicidal ideation. Bureaucratic burden, age and taking care of children are significant predictors of ERI. No significant gender differences were observed.

**Conclusions:**

ERI is prevalent among German dentists and closely linked to impaired mental health. Administrative burdens represent an important modifiable stressor. Reducing bureaucratic workload and improving structural working conditions may help preventing and restoring effort-reward balance and may contribute to reducing depressive symptoms and suicidal ideation among dentists in Germany.

## Background

Depressive symptoms and suicidal ideation are important indicators of mental health burden and occupational strain in healthcare professionals. Dentists may be particularly vulnerable because their work combines high clinical responsibility, sustained concentration, physical strain, emotional demands, and increasing administrative workload [1, 2]. In Germany, structural changes in dental care and growing bureaucratic requirements may further increase occupational stress and contribute to an imbalance between professional effort and perceived rewards [2, 3]. Furthermore, family commitments, such as childcare or caregiving responsibilities, may contribute to work-family conflict and additional stress, particularly when combined with high occupational demands [4]. Beyond these psychosocial demands, dentistry also requires profound expertise, manual skills in highly detailed working areas, physical endurance, emotional stability, and entrepreneurial competencies [1, 5].

In the present study, depressive symptoms and suicidal ideation are considered the main mental health outcomes. Depressive symptoms refer to self-reported symptoms of depressed mood and loss of interest during the previous two weeks. They are not interpreted as a clinical diagnosis of major depression, but as a screening-based indicator of current depressive symptom burden assessed with the Patient Health Questionnaire-2 (PHQ-2) [6, 7]. Suicidal ideation refers to self-reported thoughts of being better off dead or of self-harm during the previous two weeks, as assessed by item 9 of the Patient Health Questionnaire-9 (PHQ-9). Both outcomes are therefore understood as indicators of current psychological distress among dentists.

Particularly challenging are external work determinants, such as irregular working hours and lack of breaks, high noise levels, working in constrained postures, and high emotional demands [1]. It is therefore unsurprising that dentists face increasing physical and psychological strain [1]. Particularly, self-employed dentists are often perceived as highly resilient individuals with considerable composure. However, these burdens can significantly impact even the most resilient personalities in the long term, leading to exhaustion and burnout [1]. In private practices, limited exchange with colleagues may add to perceived occupational strain and feelings of professional isolation [1]. Reconciling family and career is also a significant challenge, especially for female dentists. A German study from 2019 found that 42% of female dentists reported having to choose between having a child or a career, compared to only 18% of their male colleagues. This discrepancy points to gender-specific differences in stress levels in the dental profession. The double burden of professional obligations and family responsibilities may contribute to a higher overall workload for many female dentists. In addition, women often take on care work for relatives, which may further increase physical and psychological strain [8].

A 2022 meta-analysis based on 17 international studies reported an overall burnout prevalence of 13% among dentists, with prevalence estimates ranging from 4% to 32% across individual studies [9].

A German study showed an association between higher stress levels and more severe musculoskeletal pain among the dentists surveyed [5]. Another study of 1,231 German dentists found that 60.99% of respondents (*n* = 730) rated dental practice as above-average stressful. More than half of the participants reported stress-related complaints such as listlessness, exhaustion, sleep problems, and anxiety. In addition, 32% were at risk of burnout, 44% reported depressive symptoms, and 13% reported suicidal thoughts [1].

Administrative workload represents another important stressor in German dentistry. Meyer-Theewen (2024) identified administrative activities and the associated documentation effort as a significant stress factor in dentistry in Germany [2]. Although these bureaucratic tasks are necessary from a business and organizational perspective, many dentists perceive them as incompatible with their professional self-image as medical professionals. Moreover, the time required for these tasks often reduces the time available for patient treatment or recovery [2].

Previous research therefore suggests that sociodemographic, family-related, and occupational characteristics may shape the experience of work-related stress and mental health burden. In dentistry, working hours, employment status, physical strain, organizational demands, family responsibilities, and perceived bureaucratic burden have been discussed as relevant aspects of occupational stress [1, 2, 5, 8]. Similar associations between bureaucratic workload, organizational stressors, effort-reward imbalance, burnout risk, and depressive symptoms have also been described in other healthcare professions [10, 11]. These findings support the inclusion of age, gender, working hours, self-employment, caring responsibilities, and perceived bureaucratic burden as related risk factors in the present study.

Against this background, Siegrist’s Effort-Reward Imbalance (ERI) model provides the theoretical framework for the present study. According to this model, employees expect rewards such as fair income, recognition, job security, and opportunities for professional development in exchange for their occupational efforts. A gratification crisis may occur when the balance between efforts and rewards is disrupted, which may increase the risk of mental and physical health problems in the medium and long term [12]. An ERI ratio of 1 reflects a balance between efforts and rewards; values below 1 denote a favourable situation in which rewards outweigh efforts, whereas values above 1 indicate an unfavourable imbalance associated with increased health risks [12]. Thus, not only the objective workload is relevant, but also the subjectively perceived discrepancy between effort and reward [12].

The ERI model has been validated and widely applied in occupational health research. In the international study “The Measurement of Effort-Reward Imbalance at Work: European Comparisons”, the model was tested using data from 18,643 employees from five European countries and different occupational groups, including healthcare, public, technical, and transport sectors. In 12 of the 14 analyses, high effort-reward imbalance was significantly associated with reduced self-reported health [13]. Subsequent research has established the ERI model as one of the central theoretical frameworks in psychosocial stress research, with consistent associations across a range of health outcomes, including cardiovascular disease, depressive disorders, and work disability [14–16].

Although the ERI model has been widely applied in occupational health research, evidence on effort-reward imbalance among dentists remains limited, particularly in Germany. Existing German studies indicate substantial occupational strain among dentists, but they have primarily described general burden profiles and specific stressors rather than systematically examining occupational stress within the theoretical framework of effort-reward imbalance. In German dentistry, this imbalance may be particularly relevant because dentists are confronted with clinical responsibility, economic pressure, administrative documentation duties, and expectations of high-quality patient care. From an ERI perspective, perceived rewards may also be affected by limited professional autonomy, insufficient recognition of non-clinical workload, and the experience that bureaucratic tasks reduce time for patient care and recovery [2, 12].

Depressive symptoms and suicidal ideation are therefore relevant outcomes because they reflect substantial psychological distress and may be associated with impaired quality of life and reduced professional functioning. Compared with data from the German general population, the observed prevalences of depressive symptoms and suicidal ideation among dentists appear elevated [17, 18]. Despite these indications, effort-reward imbalance, depressive symptoms, and suicidal ideation have rarely been examined together in practising dentists. Furthermore, it remains insufficiently understood which sociodemographic, family-related, and occupational factors are associated with higher effort-reward imbalance in this group. Against the background of increasing administrative demands in dentistry, perceived bureaucratic burden may be particularly relevant [2, 10, 11].

The present study addresses this research gap by examining effort-reward imbalance, related sociodemographic, family-related, and occupational risk factors, and their associations with depressive symptoms and suicidal ideation in a nationwide sample of practising dentists in Germany. Specifically, the study addresses the following research questions:

1. How pronounced is effort-reward imbalance among practising dentists in Germany?
2. Which sociodemographic, family-related, and occupational factors are associated with higher effort-reward imbalance?
3. Is effort-reward imbalance associated with depressive symptoms?
4. Is effort-reward imbalance associated with suicidal ideation?

Based on the ERI model and previous empirical findings, we formulated the following hypotheses:

H1: Effort-reward imbalance is common among practising dentists in Germany.

H2: Higher effort-reward imbalance is associated with a higher likelihood of depressive symptoms among practising dentists in Germany.

H3: Higher effort-reward imbalance is associated with a higher likelihood of suicidal ideation among practising dentists in Germany.

H4: Sociodemographic, family-related, and occupational risk factors are associated with higher effort-reward imbalance and provide additional explanatory value for depressive symptoms and suicidal ideation.

## Methods

Between July 23, 2021, and February 28, 2022, a nationwide online survey was conducted in Germany to investigate the professional activities, career aspirations, and workplace conditions of dentists. The survey was hosted on the LimeSurvey platform. To achieve the highest possible participation rate, calls for participation were disseminated through the “Zahnärztliche Mitteilungen” (ZM) journal, in cooperation with dental chambers, on websites (e.g., the Dentista e.V. homepage), newsletters, and the social media platform Instagram. Participants had to be licensed and practising dentists at the time of the survey. Ethics approval was obtained from the Ethics Committee of the Medical Faculty of Leipzig University (AZ 074/21-ek).

In total, 937 dentists participated in the survey, of which 174 dropped out right at the beginning, and another 156 participants dropped out before answering the relevant questions for the analysis presented here and were therefore excluded. Additionally, participants with incomplete age information (*n* = 14) or those who identified their gender as “diverse” (*n* = 4) were excluded, as these groups were too small for stratified gender analysis. The results presented here refer to the remaining 589 dentists.

### Instruments

The questionnaire of the entire study consisted of sections A to F, covering sociodemographic characteristics, current employment status and training, professional aspirations and expectations, current well-being, job satisfaction, attitudes toward dental care centers, and future intentions [3]. The standardized questionnaires used for the analyses presented here are described in more detail below. The PHQ-2 is an ultrashort version of the Depression Module of the Patient Health Questionnaire (PHQ-9) and serves as a screening tool for the detection of depressive disorders. It comprises the first two questions of the PHQ-9, focusing on the core criteria for major depression as defined by the Diagnostic and Statistical Manual of Mental Disorders, Fourth Edition (DSM-IV): loss of interest and depressed mood. Specifically, the PHQ-2 asks how often, over the last two weeks, individuals have been affected by little interest or pleasure in activities (Question 1) and by feeling down, depressed, or hopeless (Question 2). Response options include: “not at all,” “several days,” “more than half the days,” and “nearly every day” on a 4-point Likert scale ranging from 0 to 3. The total score of both items thus ranges between 0 and 6 [19]. Higher scores indicate more severe depressive symptoms. A score of 3 or higher indicates an increased likelihood of major depressive disorder. This cut-off point is based on receiver operating characteristic (ROC) analyses in previous validation studies. The PHQ-2 shows good to excellent psychometric properties [6, 7]. Moreover, item 9 of the PHQ-9 (‘Thoughts that you would be better off dead, or of hurting yourself in some way’) was used to assess suicidal ideation in the last two weeks. Participants were classified as having current suicidal ideation if item 9 was answered with scores from 1 (several days) to 3 (nearly every day).

Siegrist developed a standardized questionnaire with 22 items to assess effort-reward imbalance. A short 16-item form was used in this study. Each question is answered on a four-point Likert scale (1 “strongly disagree” to 4 “strongly agree”). It consists of three subscales: effort (items 1–3, e.g. “I have constant time pressure due to a heavy workload”), reward (items 4–10, e.g. “Considering all my efforts and achievements, I receive the respect and prestige I deserve at work”) and overcommitment (items 11–16, e.g. “People close to me say I sacrifice too much for my work”). For each subscale, a total score was calculated, with higher values indicating higher effort, higher reward and higher overcommitment, respectively [20, 21]. The effort-reward imbalance is calculated as follows: ERI-Ratio = $\:\frac{\mathrm{E}\mathrm{f}\mathrm{f}\mathrm{o}\mathrm{r}\mathrm{t}}{\mathrm{R}\mathrm{e}\mathrm{w}\mathrm{a}\mathrm{r}\mathrm{d}\:\mathrm{x}\:3/7}$. Higher ERI ratios indicate a stronger imbalance between effort and reward, with values above 1 indicating an unfavourable imbalance characterised by high effort relative to low reward. The ERI questionnaire has demonstrated good psychometric properties in various populations. Internal consistencies are generally acceptable to good, with Cronbach’s α ranging from 0.67 to 0.79 in samples of dental students and young dentists [3]. Test–retest reliability has been reported as high (*r* = .79–0.89) [22]. Construct validity is supported by consistent associations of high ERI ratios and overcommitment with burnout, depressive symptoms, and reduced job satisfaction, particularly among physicians and dentists. For instance, a large international study comparing physicians in Germany and Norway found that a gratification crisis (high ERI ratio) was significantly associated with lower job satisfaction in both cohorts, explaining approximately 46% of the variace in satisfaction [23]. Psychometric testing in other occupational groups and countries has consistently shown Cronbach’s α values above 0.70 for the three subscales, confirming the internal reliability of the measure [24, 25]. Longitudinal analyses have also demonstrated that ERI remains stable over time, supporting its use in prospective epidemiological research [26]. Furthermore, ERI demonstrates predictive validity beyond other established models of work stress, such as the demand-control model or organizational justice, which underscores its added value compared to other models [27]. Sociodemographic and job-related aspects were assessed with a self-developed questionnaire. Detailed information on these aspects is provided in the footnotes of the tables showing the results.

### Statistical analyses

All statistical analyses were carried out using Statistical Package for the Social Sciences (SPSS) Statistics version 29 (IBM Corp., Armonk, NY, USA). Differences between male and female participants have been tested using Chi-Square-Tests and T-tests and gender was included as a predictor variable in the regression model. To test the association between ERI Ratio and various sociodemographic, family-related and occupation factors, a linear hierarchical regression analysis was conducted, including age and gender in step 1, caring for children in the household and caring for relatives in step 2, and working hours, bureaucratic burden, and self-employment as predictors in step 3. Spearman’s rank correlations were calculated to examine the relationship between the ERI and current depressive symptoms as well as current suicidal ideation.

## Results

In the sample of 589 dentists (63.5% females; *n* = 374), 224 participants (38.0%) were under 39 years of age, 177 participants (29.8%) were between 40 and 54 years, and 188 participants (32.9%) were 55 and 77 years. Male participants were significantly older than female participants. Of the respondents, 377 (65.7%) were self-employed dentists with a significantly higher proportion of male self-employed participants (58.1%; *n* = 211 of female vs. 78.7%, *n* = 166 of male participants). Other demographic factors were also integrated into the analysis, including weekly working hours, taking care of children, and providing care for relatives. Overall, 69.3% of participants reported having children. In 41.1% of the total sample, children lived permanently in the household, in 9.7% they lived partly in the household, and in 18.5% they did not live in the household. In addition, 5.9% (*n* = 44) of respondents reported currently caring for relatives. Furthermore, the survey participants were asked about their stress caused by bureaucracy at work, current depressive symptoms, and suicidal ideation. On average, participants perceived the bureaucratic workload in their jobs as stressful (M = 4.01, SD = 1.0), on a scale ranging from 1 (‘not at all stressful’) to 5 (‘very stressful’), with higher stress levels among male participants. Female participants were less often self-employed, had fewer working hours per week, were more likely to permanently take care of children, and reported a lower burden due to bureaucracy compared to male participants (for more details, see Table 1). 21.1% (*n* = 125) of the participants were screened positive for depressive symptoms using the PHQ-2; 11.7% (*n* = 69) of respondents reported having had suicidal thoughts in the last two weeks. The mean ERI ratio is 1.1 (SD = 0.5). More than half of the respondents (53.1%) had an ERI ratio above 1, indicating an imbalance between effort and reward in a negative manner. There were no gender differences with respect to depression, suicidal ideation, effort, reward, overcommitment and the ERI ratio (for more details see Table 1).

**Table 1:** Characteristics of the study sample stratified by gender

	Female *n* = 374	Male *n* = 215	Total *n* = 589	Test for Gender^5^
age (M/ SD)	(43.92/ 11.50)	(49.88/ 11.90	(49.88/ 11.90	T=-5.97; *p*<.001
Age groups % (n) < 40 years 40–54 years 55 + years	44.70 (167) 30.40 (115) 24.23 (92)	26.50 (57) 28.91 (62) 46.26 (96)	38.00 (224) 29.81 (177) 32.89 (188)	Chi^2^=41.17,*p*<.001
self-employed % (n)^1^	58.1 (211)	78.7 (166)	65.7 (377)	Chi^2^=24.99,*p*<.001
working hours per week M (SD)	37.40 (12.77)	44.65 (12.71)	40.05 (13.20)	T=-6.65;*p*<.001
working hours per week % (n) < 35 35–45 > 45	99 (26.5) 202 (54.0) 73 (19.5)	23 (10.7) 105 (48.8) 87 (40.5)	122 (20.7) 307 (52.1) 160 (27.2)	Chi2 = 39.15,*p*<.001
Children living in the household % (n) No children Full time Partly no	34.5 (129) 43.6 (163) 6.7 (25) 15.2 (57)	24.2 (52) 36.7 (79) 14.9 (32) 24.2 (52)	30.7 (181) 41.1 (242) 9.7 (57) 18.5 (109)	Chi2 = 21.66,*p*<.001
Caring for relatives % (n)	6.1 (23)	5.1 (11)	5.9 (44)	Chi^2^=0.268, n.s.
Burden of bureaucracy^2^ M (SD)	3.88 (1.02)	4.24 (0.99)	4.01 (1.02)	T=-4.11;*p*<.001
PHQ-2 > 2% (n)	19.7 (74)	23.6 (51)	21.2 (125)	Chi^2^=1.27, n.s.
Suicidal ideation^3^% (n)	11.8 (44)	11.6 (25)	11.7 (69)	Chi^2^=362, n.s.
ERI: Effort M (SD)	8.67 (2.02)	8.95 (1.91)	8.78 (1.98)	T=-1.66; n.s.
ERI: Reward M (SD)	19.67 (4.41)	19.44 (4.43)	19.59 (4.41)	T = 0.613; n.s.
ERI: Overcommitment M (SD)	15.10 (4.00)	14.49 (4.18)	14.87 (4.07)	T = 1.76; n.s.
ERI: Ratio^4^ M (SD)	1.11 (0.49)	1.17 (0.51)	1.13 (0.49)	T=-1.29; n.s.
ERI: Ratio %(n) 0-0.8 < 0.8-1.0 < 1.0-1.5 < 1.5	23.3 (87) 25.4 (95) 35.6 (133) 15.8 (59)	20.9 (45) 22.8 (49) 37.3 (79) 19.5 (42)	22.4 (132) 24.4 (144) 36.0 (212) 17.1 (101)	Chi^2^=1.89, n.s.

^1^self-employed includes the following activities: “self-employed in own practice”, “self-employed in a group practice”, “self-employed in a group practice”, “self-employed in a supra-local group practice”.; ^2^"Do you perceive the bureaucratic workload as a burden in your profession?” 1 = “not at all”, 2 = “a little”, 3 = “somewhat”, 4 = “a lot”, 5 = “very much”; ^3^"Thoughts that you would be better off dead, or of hurting yourself in some way?” Answer options: 0 = “not at all”, 1 = “on individual days”, 2 = “on more than half of the days”, 3 = “almost every day”. Scores > 0 rated as suicidal thoughts.; ^4^ ERI-Ratio =$\:\frac{\mathrm{E}\mathrm{f}\mathrm{f}\mathrm{o}\mathrm{r}\mathrm{t}}{\mathrm{R}\mathrm{e}\mathrm{w}\mathrm{a}\mathrm{r}\mathrm{d}\:\mathrm{x}\:3/7}$

### Factors associated with effort-reward imbalance

Table 2 shows the results of the hierarchical linear regression analysis testing factors associated with effort-reward imbalance, operationalised by the ERI ratio. In the first step, age and gender were included as sociodemographic variables. Only age was a significant predictor and remained significant across all three steps of the regression analysis. Participants in the youngest age group had the lowest mean ERI ratio (M = 1.04, SD = 0.45), those in the middle age group had the highest mean ERI ratio (M = 1.21, SD = 0.53), and those in the oldest age group showed an intermediate ERI ratio (M = 1.17, SD = 0.51).

In the second step, children living in the household and caring for relatives were additionally included as family-related predictors. Children living in the household was a significant predictor and remained significant in the third step of the regression analysis. Participants whose children lived with them only part of the time had the highest ERI ratio (M = 1.25, SD = 0.51). Lower ERI ratios were found among participants without children (M = 1.16, SD = 0.49), participants whose children permanently lived in the household (M = 1.12, SD = 0.55), and participants whose children did not live in the household (M = 1.04, SD = 0.36).

In the third step, occupational predictors, including working hours, self-employment, and perceived bureaucratic burden, were included in the model. Only perceived bureaucratic burden was a significant predictor. Dentists who reported low bureaucratic burden showed a lower ERI ratio (M = 0.79, SD = 0.32), whereas those with the highest subjective bureaucratic burden showed a higher ERI ratio (M = 1.38, SD = 0.56). The sociodemographic variables in step 1 explained less than 1% of the variance. The explained variance increased to 4.4% in step 2, but remained low. The occupational predictors in step 3 substantially increased the explained variance to 18.2%.

**Table 2:** Results of the linear stepwise regression analysis for Effort-Reward Imbalance (Ratio)

	Model 1		Model 2		Model 3	
	Regression coefficient B	*p*	Regression coefficient B	*p*	Regression coefficient B	*p*
Age^0^	**0.058**	**0.022**	**0.157**	**< 0.001**	**0.076**	**0.029**
Gender^1^	0.030	0.492	0.039	0.373	-0.009	0.825
Children^2^			**-0.123**	**< 0.001**	**-0.113**	**< 0.001**
Care^3^			-0.179	0.050	-0.050	0.078
Self-employed^4^					-0.051	0.336
Working hours^5^ per week % (n)					0.053	0.086
Burden of bureaucracy^6^					**0.195**	**< 0.001**
Model	Adj. R^2^ = 0.008	*P* = .034	Adj. R^2^= 0.044	*p* < .001	Adj. R^2^= 0.182	*p* < .001

undefined^0^age groups: 1 = < 40 years; 2 = 40–54 years; 3 = 55+ years; ^1^female = 1; male = 2; ^2^0=no children; 1 = children in the household; 2 children partly living in the household; 3 = children not living in the household; ^3^yes = 1; no = 0; ^4^self-employed = 1; not self-employed = 0; ^5^working hours in 3 groups, please refer to Table 1; ^6^"Do you perceive the bureaucratic workload as a burden in your profession?” Possible answers: 1 = “not at all”; 2 = “a little”; 3 = “somewhat”; 4 = “a lot”; 5 = “very much”

### Correlation between ERI ratio, depressive symptoms, and suicidal ideation

The correlation between the ERI ratio and the PHQ-2 score is medium positive (*r* = .537, *p* < .001), indicating that dentists with a higher ERI ratio reported more severe depressive symptoms. Participants who screened positive for depressive symptoms showed significantly higher ERI ratios than those who screened negative (M^DEP^ = 1.59, SD = 0.61 vs. M^NODEP^ = 1.01, SD = 0.37; T = -13.04, *p* < .001). In addition, a moderate positive correlation was found between suicidal ideation and ERI ratio (*r* = .277, *p* < .001), indicating that a higher imbalance between effort and reward is associated with an increased probability of current suicidal ideation (M^SI^ = 1.52, SD = 0.63 vs. M^NOSI^ = 1.08, SD = 0.45; T = -7.15, *p* < .001).

## Discussion

The results show that a considerable proportion of the dentists surveyed in Germany experience an imbalance between professional effort and rewards received (effort-reward imbalance), since more than half of the sample (53.1%) has an ERI ratio above 1. The mean ratio in the sample under study was 1.1. In comparison, ERI mean scores in large representative German workforce samples ranged from 0.83 to 0.89, suggesting that dentists may experience a more pronounced effort-reward imbalance than the general working population [28]. This finding is also consistent with previous German studies that have described substantial occupational stress, burnout symptoms, physical strain, depressive complaints, and suicidal thoughts among dentists [1, 2, 5]. In our study, medium positive correlations were found between the ERI ratio and current depressive symptoms as well as suicidal ideation. This finding is in line with the assumptions of Siegrist’s ERI model and with previous empirical findings showing associations between effort-reward imbalance and adverse mental health outcomes across different occupational groups [14, 15]. A systematic review and meta-analysis of prospective cohort studies further found that effort-reward imbalance was associated with an increased risk of depressive disorders [16]. For instance, the Whitehall II study in the UK demonstrated that employees with high ERI had significantly increased risks of coronary heart disease and poorer health functioning [29]. Similarly, findings from the German SOEP and the French CONSTANCES cohort showed that psychosocial work stress and adverse employment histories are associated with sickness absence, poor self-rated health, depression, and premature exit from the labour market [30, 31]. It is particularly striking that over 21% of participants screened positive for depressive symptoms and 11.7% reported suicidal ideation in the past two weeks. Compared with data from the German general population, these prevalence rates appear elevated [17, 18]. Forkmann et al. reported a point prevalence of suicidal ideation of about 8% in the German general population, whereas in our sample 11.7% reported suicidal ideation [17]. Similarly, the proportion of participants who screened positive for depressive symptoms in our study exceeded prevalence estimates reported in German population-based studies [18]. Direct comparisons should be interpreted cautiously because our sample is not representative and there are differences to the study design of the studies investigating representative data from the general population. Nevertheless, these findings suggest that dentists may represent an occupational group with elevated mental health burden.

Three variables showed significant associations with the ERI ratio: age, children living in the household and perceived bureaucratic burden. Perceived bureaucratic burden showed the strongest association with higher ERI values in our analysis with a substantial increase in the variance explained in step 3 of the regression analysis. This finding emphasizes the importance of administrative tasks as a major stressor in the everyday work of dentists. It is consistent with earlier observations that many dentists perceive documentation workload as stressful and as poorly aligned with their professional self-understanding as medical professionals [2]. From an ERI perspective, bureaucratic tasks may increase perceived effort because they add non-clinical workload, reduce the time available for patient care, and limit opportunities for recovery. At the same time, these tasks may not be experienced as adequately rewarded, particularly if they are perceived as insufficiently recognized or incompatible with dentists’ professional identity. Comparable observations have been made in other healthcare professions, where bureaucratic workload, organizational injustice, and effort-reward imbalance have been associated with burnout risk and depressive symptoms [10, 11]. This suggests that the structural stressors observed in dentistry may reflect broader organizational challenges in healthcare.

Caring responsibilities were also associated with higher ERI values, especially among participants who reported partial childcare responsibilities in their household. One possible explanation is that shared or alternating childcare arrangements may be associated with additional organizational demands, reduced planning security, or increased coordination efforts. However, a conclusive assessment of these associations is not possible based on the available data. Further research is needed to examine how different forms of family responsibility and care work are related to effort-reward imbalance among dentists.

It was also noticeable that neither self-employment as a practice owner, working as an employee, nor working hours had a significant influence on the ERI ratio. This suggests that structural stress factors, such as bureaucracy, may be relevant across different professional positions and irrespective of working time. Surprisingly, gender had no significant influence on the ERI ratio. This finding is noteworthy because gender-related differences in family responsibilities and career constraints have been described in medical professions [8]. It is conceivable that the ERI score does not adequately reflect certain gender-specific dimensions of stress, such as work-life balance or care work.

### Limitations

Several limitations should be considered when interpreting the findings. First, the cross-sectional design does not allow conclusions about causal or temporal relationships between effort-reward imbalance, related sociodemographic, family-related, and occupational risk factors, depressive symptoms, and suicidal ideation. It therefore remains unclear whether higher ERI contributes to poorer mental health, whether depressive symptoms influence the perception of occupational effort and reward, or whether both are shaped by additional unmeasured factors. Second, all data were based on self-report measures, which may be affected by recall bias, social desirability, and subjective perception. Third, depressive symptoms and suicidal ideation were assessed using screening instruments rather than clinical diagnostic interviews. Thus, the findings should be interpreted as indicators of current symptom burden, not as clinical diagnoses. Fourth, the online survey design and the voluntary nature of participation may have introduced selection bias, as dentists with a particular interest in occupational stress or mental health may have been more likely to participate. Although the sample included practising dentists from Germany, the results may not be fully generalizable to all dentists in Germany or to dentists in other healthcare systems.

Despite these limitations, the findings are broadly consistent with international ERI research. Reviews have concluded that the ERI questionnaire is psychometrically robust across cultures, predictive of a wide range of adverse health outcomes, and applicable in both general population cohorts and occupational subgroups [32, 33]. The convergence of our results with this broader evidence base supports the relevance of the ERI framework for analysing occupational stress and mental health among dentists.

Furthermore, the relatively large nationwide sample provides an important basis for examining effort-reward imbalance and mental health outcomes among practising dentists in Germany. Future longitudinal studies are needed to investigate temporal and causal relationships between occupational stressors, effort-reward imbalance, depressive symptoms, suicidal ideation, and related sociodemographic, family-related, and occupational risk factors in a more differentiated manner.

## Conclusion

This study demonstrates that a considerable proportion of German dentists experience effort-reward imbalance (ERI), with more than half of the sample showing an ERI ratio above 1. Higher ERI is closely associated with impaired mental health, including depressive symptoms and suicidal ideation. Administrative burden emerged as the strongest predictor of increased ERI, highlighting its role as a major stressor that many dentists perceive as incompatible with their understanding of medical work. Challenges related to childcare, particularly in alternating custody arrangements, were also associated with higher ERI.

These findings suggest that structural and organizational factors may play an important role in occupational stress among dentists. Future preventive measures may therefore consider reducing bureaucratic workload, improving structural working conditions, and supporting work-life integration to promote effort-reward balance and mental health among dentists.

In conclusion, ERI represents a significant occupational health risk among German dentists. Addressing modifiable occupational stressors may contribute to improving working conditions and supporting the long-term mental health of the dental workforce.

## Data Availability

The data that support the findings of this study are not publicly available due to privacy and ethical restrictions but are available from the corresponding author on reasonable request.

## Abbreviations

ERI: Effort-Reward Imbalance
PHQ-2: Patient Health Questionnaire-2
PHQ-9: Patient Health Questionnaire-9
SPSS: Statistical Package for the Social Sciences
